# African Schistosomiasis: A Framework of Indicators Assessing the Transmission Risk and Intervention Effectiveness

**DOI:** 10.3390/tropicalmed9110275

**Published:** 2024-11-13

**Authors:** Hong-Mei Li, Nicholas Midzi, Masceline Jenipher Mutsaka-Makuvaza, Zhi-Qiang Qin, Shan Lv, Shang Xia, Ying-Jun Qian, Robert Berquist, Xiao-Nong Zhou

**Affiliations:** 1 National Institute of Parasitic Diseases, Chinese Center for Disease Control and Prevention (Chinese Center for Tropical Diseases Research), Shanghai 200025, China; lihm@nipd.chinacdc.cn (H.-M.L.); qinzq@nipd.chinacdc.cn (Z.-Q.Q.); lvshan@nipd.chinacdc.cn (S.L.); sxia@nipd.chinacdc.cn (S.X.); qianyj@nipd.chinacdc.cn (Y.-J.Q.); 2 NHC Key Laboratory of Parasite and Vector Biology, Shanghai 200025, China; 3 WHO Collaborating Centre for Tropical Diseases, Shanghai 200025, China; 4 National Center for International Research on Tropical Diseases, Shanghai 200025, China; 5 National Institute of Health Research, Ministry of Health and Child Care, Harare P.O. Box CY 573, Zimbabwe; nmidzi@mohcc.org.zw (N.M.); mascelinejeni@gmail.com (M.J.M.-M.); 6 Department of Microbiology and Parasitology, School of Medicine and Pharmacy, College of Medicine and Health Sciences, University of Rwanda, Huye, Butare P.O. Box 117, Rwanda; 7 Geospatial Health, Ingerod 407, SE-454 94 Brastad, Sweden; robert.bergquist@outlook.com

**Keywords:** schistosomiasis, influencing factors, intervention measures, indicator, Delphi method, entropy method

## Abstract

Schistosomiasis, a parasitic disease with a complex transmission mechanism, requiring a snail intermediate host, is influenced by biology, the environment, human behavior and the prevailing socioeconomic situation. This study aimed to systematically investigate the importance and feasibility of indicators related to the factors influencing transmission and intervention measures for *Schistosoma mansoni* and *S. haematobium*. Based on a literature review and group discussions according to the Delphi method, a framework questionnaire was designed. A total of 33 experts on schistosomiasis were invited, and 27 were accepted, to rate the importance and feasibility of indicators for transmission with and the control of schistosomiasis, with a focus on intervention measures for *S. mansoni* and *S. haematobium* infections in Zimbabwe. After two rounds of Delphi consultations with these experts, calculated to have a high average authority coefficient (0.88), a consensus was reached on a framework that included 2 primary, 6 secondary and 39 tertiary indicators. The Delphi–entropy method was applied to assess the weight of each indicator. The key influencing factors included hazardous water exposure, accessibility to safe drinking water, sanitary facilities and the contamination of water bodies by outdoor defecation/urinary habits. The intervention measures involved improved diagnostics, health education, preventive chemotherapy, the presence of national control plans and the implementation of the strategy on water, sanitation and hygiene (WASH). While these factors are already well known, their detailed order of importance could help to improve the allocation of specific control efforts.

## 1. Introduction

Schistosomiasis is caused by parasitic worms and is one of the neglected tropical diseases (NTDs) identified by the World Health Organization (WHO) [1,2,3]. Five species of schistosomes parasitize humans, namely *Schistosoma mansoni*, *S. haematobium*, *S. intercalatum*, *S. japonicum* and *S. mekongi*. The first three species are prevalent in Africa, with *S. mansoni* also present in Latin America [1,2,3]. *S. japonicum* is confined to China, the Philippines and Sulawesi Island of Indonesia, while *S. mekongi* exists only in pockets along the Mekong River, where it traverses the border between Cambodia and the Lao PDR [1,4]. Importantly, more than 90% of the total burden of the disease is confined to sub-Saharan Africa, where schistosomiasis is endemic in 51 out of 54 African countries [5,6].

*S. mansoni*, *S. haematobium* and *S. intercalatum* rely exclusively on humans as definitive hosts, while *S. japonicum* and *S. mekongi* also infect in a variety of other mammals. Various species of freshwater snails act as intermediate hosts—generally, *Biomphalaria* spp. for *S. mansoni* and *S. intercalatum*, *Bulinus* spp. for *S. haematobium*, *Oncomelania* spp. for *S. japonicum* and *Neotricula* spp. for *S. mekongi* [1,2,3,4]. The definitive host excretes parasite eggs with feces or urine (depending on species), and hatched eggs release miracidia that develop into sporocysts in infected snails, which eventually discharge cercariae into the surrounding water. People are easily infected when in contact with contaminated water and finally come to harbor schistosomula that grow into adult worms capable of egg production [7]. The continuation of this cycle depends specifically on various factors that not only include natural environmental conditions, such as the temperature and rainfall, which mainly act on the snail populations [8,9,10], but also on the demography, socioeconomy and human behavior [11]. Endemicity is also linked to geography, not only depending on the temperature but also on the presence of water bodies, e.g., there is a higher incidence of schistosomiasis in populations living close to natural water sources [12,13], while features such as the elevation and slope also impact the distribution [14,15]. Hydrology, such as the construction of irrigation projects, reservoirs and dams, affects the incidence of schistosomiasis directly [16,17]. Human activities associated with fishing, farming, washing and swimming, as well as a lack of safe drinking water and sanitation, accelerate the spread of schistosomiasis [18]. Africa’s limited sanitation infrastructure and low socioeconomic levels exacerbate this vicious cycle [19].

Preventive chemotherapy with praziquantel, which is the only available drug against schistosomiasis at the moment [20], has played a significant role in reducing the number of schistosomiasis cases in Africa [21,22,23]. However, its lack of effect against juvenile schistosomes, coupled with insufficient drug coverage, has contributed to the continued high prevalence of schistosomiasis in Africa [24]. The WHO’s goal to eliminate schistosomiasis as a public health problem by returning to the old focus on interrupting transmission [25] presents both opportunities and challenges for schistosomiasis control programme, especially in Africa. In 2022, the WHO Global Strategy for Schistosomiasis Control and Elimination [25] recommended setting infection rate thresholds, expanding preventive chemotherapy based on mass drug administration (MDA) and implementing the water, sanitation and hygiene (WASH) strategy, as well as snail control. These approaches, together with improved diagnostic tools and cross-sector collaboration, should reach this goal, provided that sufficient resources and funding for schistosomiasis control are obtained.

While the risk factors and effectiveness of intervention measures have been studied in detail, few authors have systematically analyzed the various variables involved in the transmission and control of the parasite, including their relative importance and feasibility [26]. Investigations of the comparative influences of the climate, natural environment, biological factors and human behavior should not only contribute to a detailed understanding of the disease transmission but also contribute to the exploration of more effective intervention measures and how they can be implemented together.

The Delphi method relies on multiple rounds of expert consultations to reach consistent and reliable expert opinions and is widely used in predicting public health trends, developing indicator systems, reaching a consensus on complex issues and conducting decision analysis [27,28,29]. It is a subjective evaluation based on expert authority and the relative importance of indicators, while the entropy method [28] assists in the calculation of the objective weights of each indicator based on the indicators’ variability. The combination of the two methods integrates subjective expert evaluation with an objective, data-driven approach. We planned to carry out a combinatory exercise focusing on *S. mansoni* and *S. haematobium* based on a literature review, group interviews, expert consultations and the combined Delphi–entropy methodology. The overall aim was to develop a comprehensive and evidence-based framework to assess the transmission risk and intervention effectiveness, allowing us to provide recommendations for policymakers and stakeholders so as to enhance the control and eventual elimination of schistosomiasis in this region.

## 2. Materials and Methods

### 2.1. Reference Literature Review

A literature search was conducted through the PubMed, Web of Science and African Journal Online databases from the beginning of records until 14 September 2023, based on the following keywords: “Africa”, “schistosomiasis”, “*Schistosoma mansoni*”, “*Schistosoma haematobium*”, “risk factor”, “influencing factor”, “intervention”, “control measure”, “system dynamics” and “transmission dynamics”. Only articles published in English and articles directly addressing the influencing factors and intervention measures for *S. mansoni* and *S. haematobium* were included. Studies on other species, those not carried out in Africa and those not published in peer-reviewed journals, e.g., conference abstracts, were excluded. A total of 183 articles were retrieved. After reviewing the titles and abstracts to exclude irrelevant studies, key publications were selected, from which we extracted indicators related to the influencing factors and intervention measures for *S. mansoni* and *S. haematobium*.

### 2.2. Group Interviews

Using the opportunity provided by the China–Zimbabwe Schistosomiasis Control Cooperation Project undertaken by the National Institute of Parasitic Diseases (NIPD) at the Chinese Center for Disease Control and Prevention (China CDC) and the National Institute of Health Research (NIHR) in Zimbabwe, a baseline field survey was conducted in the region of Shamwa, Zimbabwe. Group interviews were conducted to obtain qualitative insights into the current situation, transmission risk factors, existing control measures and recommended strategies for schistosomiasis in Zimbabwe. The interviewees included health officials and technical personnel involved in schistosomiasis control management in Mashonaland Central Province, Shamwa District, Chevakadzi Ward in Zimbabwe. The group interviews were led by two Zimbabwean project staff members, with two Chinese staff members providing technical guidance. Additionally, Chinese technical personnel from the Zimbabwean Schistosomiasis Control Cooperation Project were also interviewed. After the group interviews, the necessary information from the interview recordings was promptly extracted and transcribed into British English, from which the relevant indicators were obtained.

### 2.3. Questionnaire Design

Summarizing and consolidating the results from the literature review and group interviews, key indicators related to schistosomiasis transmission and control were extracted. After discussions with four senior schistosomiasis experts, a preliminary framework for the questionnaire on influencing factors and intervention measures for *S. mansoni* and *S. haematobium* was drafted. This framework was then further refined into a structured questionnaire survey consisting of four parts: (1) informed consent form; (2) questionnaire filling instructions; (3) core part of the expert consultation on indicators for influencing factors and intervention measures, using a 5-point Likert scale to evaluate the importance and feasibility of each indicator, as well as collecting opinions on the addition and deletion of indicators, including suggestions; and (4) personal information about the experts (name, age, gender, education, nationality, field of expertise, years of experience, etc.) and their familiarity with schistosomiasis. The selection of each indicator was based on intuition (where explicit knowledge or data were lacking), the reference literature, theoretical knowledge and practical experience. The degree of familiarity and judgment influence was divided into five levels, with each choice corresponding to a different score, the maximum of which was less than 1. Once the questionnaire had been completed, it was converted into an online format using the “Wenjuanxing” platform (https://www.wjx.cn, accessed on 25 September 2023).

### 2.4. Expert Questionnaire Consultation

To ensure the reliability of the consultation results, 33 experts were selected (and 27 accepted) from various sources, such as management personnel, technical specialists and evaluation experts from the China–Zimbabwe Schistosomiasis Control Cooperation Project, as well as a few international experts with extensive experience in schistosomiasis control.

The 27 experts were invited to participate in the questionnaire survey, either through paper questionnaires or online questionnaire links via email. The feedback from the questionnaires was promptly collected and summarized. A modified two-round Delphi expert consultation approach was employed. The first round focused on evaluating and supplementing the initial indicator framework and assessed the importance and feasibility of the indicators. Based on the results of the first round, the content of the indicator framework was adjusted, with the second round of the survey used to score the importance and feasibility of the adjusted indicators.

The expert opinions were categorized into three levels based on the five-point Likert scale, where 5 represented the highest level of importance/feasibility and 1 the opposite, i.e., no interest in or knowledge of the issues. Further, all scores above 3 indicated clear importance, while those scored below 3 were seen as lacking substance and therefore relatively unimportant. A score = 3 was an intermediate result but was clearly without strength. A consensus was considered to have been reached when more than 70% of the experts agreed, thereby making the indicators eligible for inclusion. Indicators could still be suggested for addition in the second round if 30% or more of the experts recommended them in the first round. However, if they failed after two rounds of surveys, they were excluded and not voted on again.

### 2.5. Statistical Analysis

Data were entered using Microsoft Excel and the statistical analysis was performed using IBM SPSS Statistics for Windows, version 26.0, and the R software (R Foundation, version 4.3.2). Following the Delphi method, an analysis of the experts’ basic information was conducted via the calculation of coefficients for judgment (Ca), familiarity (Cs), and authority (Cr). The latter (Cr) consisted of two parts, Ca (Table 1) and Cs (Table 2), and set with regard to how closely the indicators were related to the reliability of the evaluation results [28,30]. Cr was calculated as the arithmetic mean of Ca and Cs, i.e., Cr = (Ca + Cs)/2, with outcomes ≥0.7 supporting the authority of the expert opinion.

Kendall’s W, a non-parametric statistic for rank correlation, was used to represent the degree of consistency among the expert ratings, and the significance of the coordination coefficient was tested using the chi-square test. Statistical significance for the consistency of the expert opinions was set at *p* < 0.05. The arithmetic mean, standard deviation (SD) and coefficient of variation (CV) were calculated for the importance and feasibility ratings of each indicator. The experts were divided into groups by nationality: Zimbabwean, Chinese and other international experts. The arithmetic mean and SD of the indicators were calculated separately for each expert group. Each expert’s ratings were weighted according to their authority coefficient, and the weighted average scores for the importance and feasibility of each indicator were computed. The sum of the weighted average importance and feasibility scores for each indicator was calculated as the overall score for that indicator.

### 2.6. Indicator Weight Determination

The Delphi method is a subjective, expert opinion evaluation approach that leverages experts’ theoretical knowledge and practical experience to guide relevant research and achieve a collective judgment with concentrated opinions [27,31]. After optimizing the indicators through two rounds of consultations, the weighted importance and feasibility scores of each level of indicators were normalized. An observation matrix with *n* × *m* data points was formed by *n* consulting experts rating *m* indicators. The normalized weights of each level of indicators were then calculated with the following formula, which represents the weighted importance/feasibility score given by the *i*th expert for the *j*th indicator:

$$w_{dj}=\frac{\frac{\sum_{i=1}^{n}x_{ij}}{n}}{\frac{\sum_{j=1}^{m}\sum_{i=1}^{n}x_{ij}}{j\cdot n}}\;(i=1,2,\ldots ,n;j=1,2,\ldots ,m)$$

where *w_dj_* is the normalized weight of the *j*th indicator calculated by the Delphi method; *x_ij_* is the weighted importance/feasibility score given by the *i*th expert for the *j*th indicator with *i* (1, 2, …, *n*) and *j* (1, 2, …, *m*); *n* is the number of experts scoring each indicator; and *m* is the total number of indicators. The larger the value of *w_dj_*, the greater the importance or feasibility value of that indicator.

Weight determination by the entropy method:

The entropy method is an objective weighting method that determines indicator weights based on the amount of objective information that each indicator provided [28,32]. Based on this method, the greater the variation in the indicator values, the more information the indicator provided. This leads to a larger weight in the evaluation process; conversely, if the variation in the indicator values is smaller, the weight assigned to that indicator would be smaller. The entropy weights at each indicator level were calculated as follows.

(1) Standardization of the raw data, where *y_ij_* is the standardized weighted importance/feasibility score given by the *i*th expert for the *j*th indicator. Based on the attributes of the indicators, positive and negative indicators were standardized using Formulas (1) and (2), respectively. For positive indicators, higher values lead to better outcomes, whereas, for negative indicators, lower values are preferred.
  (1) $$y_{ij}=\frac{x_{ij}-\min x_{j}}{\max x_{j}-\min x_{j}}\;\;\;(i=1,2,\ldots ,n;j=1,2,\ldots ,m)$$
  (2) $$y_{ij}=\frac{\max x_{j}-x_{ij}}{\max x_{j}-\min x_{j}}\;\;\;(i=1,2,\ldots ,n;j=1,2,\ldots ,m)$$
(2) Calculation of the proportion of each indicator, i.e., *p_ij_* is the proportion of the *i*th sample under the *j*th indicator.
  $$p_{ij}=\frac{y_{ij}}{\sum_{i=1}^{n}y_{ij}}\;(i=1,2,\ldots ,n;j=1,2,\ldots ,m)$$
(3) Calculation of the entropy value of the indicator, i.e., *e_j_* is the entropy value of the *j*th indicator.
  $$e_{j}=-k\sum_{i=1}^{n}p_{ij}\ln\left(p_{ij}\right)\;(j=1,2,\ldots ,m;k=\frac{1}{\ln\left(n\right)})$$
(4) Calculation of the information entropy redundancy, i.e., *d_j_* is the information entropy redundancy of the *j*th indicator.
  $$d_{j}=1-e_{j}\;(j=1,2,\ldots ,m)$$
(5) Calculation of the weight of each indicator, i.e., *w_ej_* is the entropy weight of the *j*th indicator.
  $$w_{ej}=\frac{d_{j}}{\sum_{j=1}^{m}d_{j}}\;(j=1,2,\ldots ,m)$$

Weight determination by combining Delphi and the entropy method:

The comprehensive weight was calculated by combining subjective and objective weights, which reflected the subjective judgments of the experts with the intrinsic information of the indicators. The formula for the calculation of the composite weight was the following [32]:

$$w_{j}=\frac{\sqrt{w_{dj}\cdot w_{ej}}}{\sum_{j=1}^{m}\sqrt{w_{dj}\cdot w_{ej}}}\;\;\;\;\;(j=1,2,\ldots ,m)$$

where *w_dj_* represents the normalized weight obtained using the Delphi method; *w_ej_* is the weight obtained using the entropy method; and *w_j_* is the composite weight obtained by integrating both the subjective and objective approaches.

## 3. Results

### 3.1. Group Interview Responses

A total of 36 experts, i.e., 30 Zimbabwean health officials and technical personnel specializing in schistosomiasis and six Chinese schistosomiasis technical experts who had experience working in Zimbabwe, participated in the group interviews, which provided insights into the risk factors for schistosomiasis transmission in Zimbabwe, the current state of control efforts and the estimated need for further control measures. The detailed interview responses are presented in Table 3.

### 3.2. Reliability of the Expert Consultation on the Questionnaire

Out of the 33 experts invited, 27 (81.8%) accepted the first round and 24 (88.9%) also the second round. The basic information about the experts is shown in Table 4. Among the 27 experts, 15 were from Zimbabwe, 9 from China, 2 from the United Kingdom and 1 from Sweden. The average number of years of experience among the experts was 19.5 (±10.7). Nineteen (70.4%) experts held a Master’s degree or higher, 14 (51.9%) were directly involved in schistosomiasis control, 4 were engaged in global health and research related to schistosomiasis and 5 were working in nursing.

The average authority coefficient of the experts for both rounds of the questionnaire was 0.88 (Table 5), demonstrating that the selected experts had high authority in the content areas of this study and that the reliability of the study’s scoring results was high.

As seen in Table 6, the expert coordination Kendall’s W coefficients for the two rounds of expert consultation were 0.30 (χ^2^ = 736.685, *p* < 0.001) and 0.32 (χ^2^ = 722.202, *p* < 0.001), respectively, indicating that the statistical test results demonstrated consistency among the experts’ opinions and that there was a high level of agreement on the importance of the indicators.

### 3.3. Adjustment and Optimization of the Indicators

The purpose of this study was to identify the influencing factors and intervention measures for both *S. mansoni* and *S. haematobium*. Therefore, influencing factors and intervention measures were designated as the primary indicators. The key indicators were extracted based on a combination of group interviews, a literature review and expert recommendations. This made it possible to establish an initial framework of 2 primary, 6 secondary and 59 tertiary indicators related to the transmission and control of the two schistosome species.

**Table 6:** Degree of agreement of expert opinions for two rounds of consultation.

Indicator (Type)	First-Round Consultation				Second-Round Consultation			
	Indicator (No.)	Kendall’s W	χ^2^	*p*-Value	Indicator (No.)	Kendall’s W	χ^2^	*p*-Value
Influencing factors	31	0.260	349.205	<0.001	29	0.278	379.801	<0.001
Intervention measures	28	0.333	476.707	<0.001	18	0.426	358.005	<0.001
Total	59	0.300	736.685	<0.001	47	0.324	722.202	<0.001

Following the consistency results from the first round of expert importance ratings, a consensus was reached on 53 indicators, while six of them did not result in a consensus in the first round and required a second round of consultation. No additional indicators were found. Seventeen experts suggested revisions, leading to the merging of similar indicators and the removal of 13 redundant ones. In the second round, the experts evaluated the importance and feasibility of the final framework, including 2 primary, 6 secondary and 46 tertiary indicators. A consensus (average score ≥ 4) was reached on 2 primary, 6 secondary and 39 tertiary indicators. However, seven indicators did not result in a consensus, including those on religious beliefs, gender, the human impact index (HII) [12], the risk for hybridization by animal-specific and human-specific schistosome species, the normalized difference vegetation index (NDVI), elevation and slope. This suggests that the experts had differing opinions on the roles of these seven indicators in schistosomiasis transmission, indicating the need for individualized analysis.

### 3.4. Importance and Feasibility Weights of Indicators

**Figure 1:**
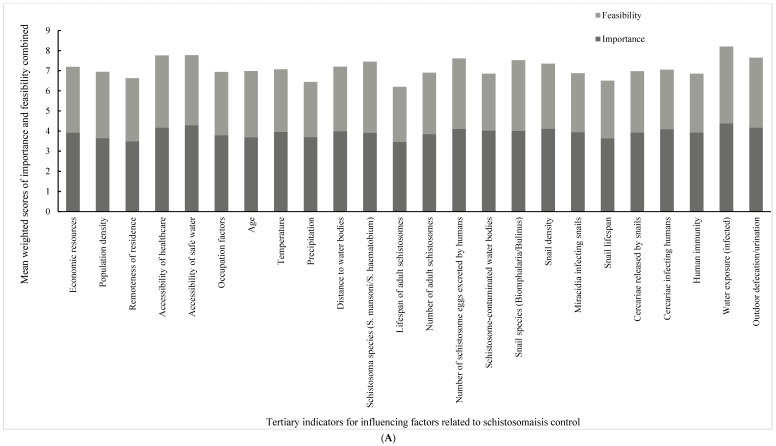

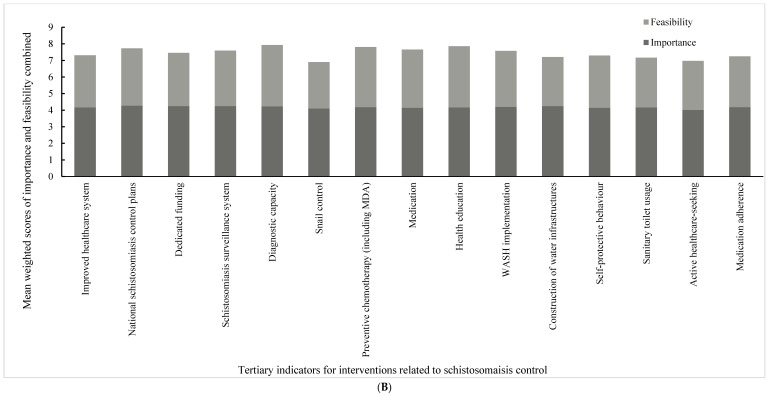
The combined weighted mean scores of importance and feasibility for each tertiary indicator. (**A**) Tertiary indicators for influencing factors related to schistosomiasis control; (**B**) tertiary indicators for interventions related to schistosomiasis control.

Table 7 and Table 8 display the importance and feasibility for each indicator agreed upon after the two rounds of expert consultation. Each table contains the mean and SD for the importance or feasibility scores of the experts from the different countries, as well as variations and weights calculated using the Delphi method, the entropy method and the combination of both for each indicator. Figure 1 ranks the combined, weighted mean scores of importance and feasibility for each indicator. 

From the comprehensive overview of the weights in Table 7 and Table 8, several were found to be the most important secondary indicators of schistosomiasis transmission. When ranked from highest to lowest importance, the feasibility of these secondary indicators was somewhat different: personal behavior, biology, the natural environment and socioeconomic factors. For intervention measures, the most important ones among the secondary indicators were found to be administrative-level interventions, followed by individual-level interventions.

The top six tertiary indicators of importance associated with transmission (Table 7) were water exposure, access to safe water, outdoor toilet facilities, access to sanitation and the human excretion of schistosome eggs. The most important intervention measures for control were dedicated funding, improved diagnostics, national control plans, surveillance systems, drug distribution and improved water infrastructure.

With regard to feasibility, the top six tertiary weight indicators associated with transmission (Table 8) were water exposure, the human excretion of schistosome eggs and the species involved, as well as access to sanitation facilities and outdoor defecation/urination habits, both with regard to the parasite and its intermediate snail host. The most feasible intervention measures for the control of both *Schistosoma* species investigated included improved diagnostics, health education, preventive chemotherapy (including MDA), drug distribution, national schistosomiasis control plans and WASH implementation.

## 4. Discussion

Preventive chemotherapy with praziquantel is the main intervention recommended by the WHO and has played an important role in controlling schistosomiasis epidemics in Africa over the past two decades [23]. This study combined the strengths of a literature review, group interviews and expert consultations to systematically examine the key factors associated with schistosomiasis transmission and control interventions in Zimbabwe. The final, integrated framework of evaluation indicators, established after two rounds of expert consultations linked to the transmission of *S. mansoni* and *S. haematobium*, indicated that a large number of factors play a role, with personal behavior and several biological variables emerging as the most important factors in the transmission and control of schistosomiasis.

It should be noted that as many as seven indicators did not result in an expert consensus: three associated with the natural environment (elevation, slope and NDVI), one biological factor (the hybridization risk) and three socioeconomic ones (religious beliefs, gender and HII). This was due to the significantly diverse opinions presented by the experts, with some of them considering these factors to be important, while others disagreed strongly. Although they were excluded due to a lack of consensus, this does not imply that they completely lack practical significance. Indeed, there is evidence that the elevation, NDVI, slope and HII are associated with schistosomiasis prevalence [12,14,15], and the potential for hybridization to spread schistosomiasis beyond its original geographical boundaries has been demonstrated [33]. While age was agreed upon as an important indicator in this study, gender was not. However, these two areas have not been sufficiently researched and attention should be paid to the fact that women have a high water exposure rate, related to their occupational commitment to washing clothes and food preparation. We also noted that the Zimbabwean experts gave a high score to religious beliefs, while the Chinese and other international experts not. Religion is nearly absent as a determinant factor in China but remains highly relevant in many parts of Africa. Experts from Zimbabwe may emphasize the role of religion in health-seeking practices and intervention acceptance. These cultural and societal differences may affect how experts prioritize these factors.

This study found that water exposure, outdoor toilet facilities and access to sanitation were the influencing factors in the transmission of schistosomiasis, which is similar to the results of a previous study in Zimbabwe, where frequent contact with unprotected water sources, non-use of the toilet and a lack of information on schistosomiasis were found to be risk factors for schistosomiasis infection [34,35]. This study also pointed to improved diagnostic capacities and the implementation of preventive chemotherapy as feasible measures for the control of schistosomiasis [36]. The latest studies on the application of diagnostic tests for schistosomiasis in Africa have shown DNA techniques to be more promising than traditional methods (e.g., Kato-Katz thick smear or urine microscopy) thanks to their increased sensitivity [37]. Indeed, an improved diagnostic capacity is essential for disease surveillance, control and elimination [38].

Not only does the importance of the indicators vary, but the potential to control the activities that they are associated with differs as well [39,40]. This makes it difficult to control all possible influencing factors, e.g., the temperature and precipitation play indirect, albeit still critical, roles in schistosome development through their effects on the intermediate host snail populations [41,42]. In contrast, water exposure, be it swimming, bathing, playing or washing clothes, can theoretically be controlled, as can the number of schistosome eggs from humans reaching the intermediate host. In the latter case, better drug distribution and the prevention of outdoor defecation/urination would have a direct effect as both are closely aligned with the parasite’s life cycle. This suggests that schistosomiasis control efforts should focus on factors playing key roles in transmission, as they represent feasible and cost-effective strategies in the control of schistosomiasis.

Among the experts consulted in this study, 21 (78%) had practical experience in schistosomiasis control in Zimbabwe, providing a solid foundation for subsequent studies on the transmission factors, control strategies and intervention models in a typical sub-Saharan, endemic country. Schistosomiasis has long been neglected in Zimbabwe, with the primary control strategy based on MDA with praziquantel targeting school-aged children, as recommended by the WHO [43]. We also observed differences in the importance and feasibility ratings of the indicators among the experts, e.g., the Zimbabwean experts identified the following as the five most useful measures: preventive chemotherapy by MDA, health education, improved diagnostics, better-targeted national control programs and chemotherapy for all in need of it. The Chinese experts had a similar list: preventive chemotherapy by MDA, health education and improved diagnostics, the implementation of the WASH strategy and surveillance systems were the most feasible interventions. Meanwhile, the other international experts regarded health education, improved diagnostics, national control plans, preventive chemotherapy by MDA and the implementation of WASH as the interventions of choice. Given the complexity of schistosomiasis transmission, it is necessary to focus on factors for its control that can be modified. The differences in the importance and feasibility ratings provided by experts from different countries offer valuable insights for the selection of appropriate intervention strategies moving forward.

Among the limitations of this study, we identified the diverse geographical locations of the experts, which necessitated the use of online questionnaires, an aspect that might have impacted the response rates negatively. Furthermore, since most experts were from Zimbabwe, their specific knowledge and experience may have influenced the applicability of the indicators more than those of the other experts. Future research could benefit from involving a broader range of international experts to improve the discussion on which indicators to apply. The third limitation was the absence of community-level data, which could present a more accurate reflection of the local situation. Future studies incorporating such data will be crucial in validating the framework and providing detailed insights into schistosomiasis transmission and control.

## 5. Conclusions

This study employed a combination of a literature review, group interviews, a Delphi expert consultation and the entropy method to form a consensus on a framework of evaluation indicators including influences and interventions with respect to the transmission and control of schistosomiasis. All involved factors interacted, forming an integrated framework of evaluation indicators consisting of 2 primary, 6 secondary and 39 tertiary indicators, 24 of which were related to influencing factors and 15 to intervention measures. While the key influencing factors in the transmission of *S. mansoni* and *S. haematobium* included all forms of water exposure, access to safe drinking water, access to sanitation facilities, outdoor defecation/urination habits and the number of parasite eggs excreted by humans, the most feasible intervention measures were found to be better diagnostic capabilities, health education, preventive chemotherapy (especially MDA), national control plans and WASH implementation.

## Figures and Tables

**Table 1:** Variation in the expert judgment coefficient.

Judgment Criterion	Degree of the Judgment’s Influence				
	High	Relatively High	Moderate	Relatively Low	Low
Theoretical knowledge	0.3	0.25	0.2	0.15	0.1
Practical experience	0.5	0.45	0.4	0.35	0.3
References	0.1	0.1	0.1	0.1	0.1
Intuition	0.1	0.1	0.1	0.1	0.1

**Table 2:** Variation in the expert familiarity coefficient.

Level of Familiarity with Schistosomiasis	Degree of Familiarity
Very familiar	0.900
Quite familiar	0.675
Moderately familiar	0.450
Slightly familiar	0.225
Not at all familiar	0

**Table 3:** Key points from the group interviews.

Question	Response
Q1: Status of schistosomiasis in public health in Zimbabwe?	Although neglected for a long time, schistosomiasis is a significant public health issue in Zimbabwe. In the latest national health strategy, it is listed as number 4 in importance after HIV, malaria and tuberculosis. However, there is no dedicated disease control system or institution for schistosomiasis in Zimbabwe, so control efforts are integrated into the general hospital management system, following a unified medical and preventive management model.
Q2: Risk factors for schistosomiasis transmission in Zimbabwe?	A basic understanding of schistosomiasis transmission exists as identified through the following risk factors. (1) The greatest risk factor is water contact. Those women in common occupational contact with water and also children are at high risk. (2) Lack of disease awareness without practice of protective measures. (3) Shortage of schistosomiasis testing methods and equipment, with diagnosis only based on clinical symptoms (e.g., blood in urine). (4) Schistosomiasis screening not included in primary healthcare. (5) Insufficient supply of praziquantel. (6) Limited funding for schistosomiasis control. (7) Outdoor defecation and urination habits contribute to transmission. (8) Religious beliefs prevent some individuals from seeking treatment. (9) Lack of snail detection, monitoring tools and molluscicides. No data on snail distribution. (10) Heavy rains during the rainy season promote snail reproduction, followed by the scarcity of water during the dry season, forcing people and animals to share water sources. (11) Safe water sources are scarce, with access to drinking water from wells or communal taps among less than half of all households. (12) Rapid reinfection.
Q3: What roles do water infrastructure projects play in schistosomiasis control?	Both *Biomphalaria* and *Bulinus* snails are aquatic and difficult to control. While water infrastructure projects can improve the environment, they also increase the risk of schistosomiasis transmission. The construction of dams and large-scale water infrastructure might expand the snail distribution.
Q4: What are the current schistosomiasis control measures in Zimbabwe?	Due to budget constraints, Zimbabwe is currently mainly implementing MDA targeting school-aged children as the sole control approach.
Q5: What are your recommendations for schistosomiasis control?	The obtained recommendations for schistosomiasis control are as follows. (1) Increased attention and investment needed for schistosomiasis control. (2) National schistosomiasis control strategies and management guidelines would be of important assistance. (3) Given the lack of infrastructure, safe drinking water and sanitation facilities, including a WASH strategy, are critically needed. (4) Training and capacity building for personnel need strengthening. (5) Accurate and rapid disease detection technologies required to improve detection rates. (6) Snail control should be implemented in parallel with MDA. (7) Health education efforts should be enhanced, with more health education products and protective equipment provided. (8) More drug resources needed.

HIV = human immunodeficiency virus; WASH = water, sanitation and hygiene; MDA = mass drug administration.

**Table 4:** Basic information about the consulting experts involved.

Characteristic	First-Round Consultation		Second-Round Consultation	
	Number (n)	Percentage (%)	Number (n)	Percentage (%)
Gender				
Male	20	74.1	19	79.2
Female	7	25.9	5	20.8
Age				
<40 years	4	14.8	4	16.7
40~49 years	12	44.4	9	37.5
50~59 years	9	33.3	9	37.5
>60 years	2	7.4	2	8.3
Years of experience				
<10 years	4	14.8	1	4.2
10~20 years	14	51.9	14	58.3
>20 years	9	33.3	9	37.5
Educational background				
Bachelor or student	8	29.6	5	20.8
Master	8	29.6	8	33.3
MD/PhD	11	40.7	11	45.8
Field of expertise				
Specific control	14	51.9	13	54.2
Global health	4	14.8	4	16.7
Pathogen biology	4	14.8	3	12.5
Doctor or nurse	5	18.5	4	16.7
Country				
Zimbabwe	15	55.6	12	50.0
China	9	33.3	9	37.5
Other *	3	11.1	3	12.5

* United Kingdom or Sweden.

**Table 5:** Expert authority coefficient.

Expert Source	First-Round Consultation			Second-Round Consultation		
	Ca	Cs	Cr	Ca	Cs	Cr
Zimbabwe	0.94	0.87	0.91	0.95	0.88	0.91
China	0.93	0.75	0.84	0.91	0.73	0.82
Other	0.88	0.90	0.89	0.90	0.90	0.90
Total	0.93	0.83	0.88	0.93	0.83	0.88

Ca = judgment coefficient; Cs = familiarity coefficient; Cr = authority coefficient.

**Table 7:** The most important weights for the influence and intervention indicators.

Indicator	Mean of Importance Score (±SD)				Coefficient of Variation	Delphi Weight	Entropy Weight	Comprehensive Weight
	Zimbabwean Experts	Chinese Experts	Other Inter-National Experts	All Experts				
Primary indicators								
Influencing factors	4.833 (±0.389)	4.889 (±0.333)	5.000 (±0.000)	4.875 (±0.338)	0.069	0.502	0.508	0.5050
Intervention measures	4.833 (±0.389)	4.778 (±0.441)	5.000 (±0.000)	4.833 (±0.381)	0.079	0.498	0.492	0.4950
Secondary indicators								
Socioeconomic factors	4.750 (±0.452)	4.556 (±0.527)	4.000 (±0.000)	4.583 (±0.504)	0.110	0.164	0.172	0.1675
Natural environment	4.750 (±0.452)	4.667 (±0.500)	5.000 (±0.000)	4.750 (±0.442)	0.093	0.169	0.170	0.1696
Biological factors	4.917 (±0.289)	4.667 (±0.500)	4.667 (±0.577)	4.792 (±0.415)	0.087	0.171	0.166	0.1684
Human behavioral factors	4.833 (±0.389)	4.778 (±0.441)	4.667 (±0.577)	4.792 (±0.415)	0.087	0.171	0.168	0.1694
Administrative-level interventions	4.667 (±0.492)	4.667 (±0.707)	4.667 (±0.577)	4.667 (±0.565)	0.121	0.166	0.166	0.1657
Individual-level interventions	4.667 (±0.888)	4.222 (±0.667)	4.333 (±0.577)	4.458 (±0.779)	0.175	0.160	0.159	0.1593
Tertiary indicators								
Economic resources	4.500 (±0.905)	4.333 (±0.707)	4.667 (±0.577)	4.458 (±0.779)	0.175	0.025	0.026	0.0255
Population density	4.500 (±0.798)	3.667 (±0.707)	4.000 (±1.000)	4.125 (±0.850)	0.206	0.023	0.025	0.0242
Remoteness of residence	4.000 (±1.595)	3.667 (±0.866)	4.667 (±0.577)	3.958 (±1.268)	0.320	0.022	0.022	0.0223
Accessibility of healthcare	4.750 (±0.452)	4.667 (±0.500)	5.000 (±0.000)	4.750 (±0.442)	0.093	0.027	0.026	0.0263
Accessibility of safe water	5.000 (±0.000)	4.667 (±0.500)	5.000 (±0.000)	4.875 (±0.338)	0.069	0.027	0.026	0.0269
Occupational factors	4.833 (±0.389)	3.778 (±0.667)	3.667 (±0.577)	4.292 (±0.751)	0.175	0.024	0.025	0.0245
Age	4.750 (±0.452)	3.667 (±1.118)	3.667 (±0.577)	4.208 (±0.932)	0.221	0.024	0.025	0.0241
Temperature	4.750 (±0.452)	4.222 (±0.833)	4.333 (±1.155)	4.500 (±0.722)	0.161	0.025	0.025	0.0249
Precipitation	4.167 (±1.337)	4.222 (±0.833)	4.333 (±1.155)	4.208 (±1.103)	0.262	0.024	0.024	0.0237
Distance to water bodies	4.750 (±0.452)	4.222 (±0.667)	4.667 (±0.577)	4.542 (±0.588)	0.130	0.025	0.025	0.0252
Schistosoma species (*S. mansoni*/*S. haematobium*)	4.583 (±0.793)	4.444 (±0.726)	4.000 (±1.000)	4.458 (±0.779)	0.175	0.025	0.025	0.0251
Lifespan of adult schistosomes	4.250 (±1.215)	3.778 (±1.641)	3.000 (±1.000)	3.917 (±1.381)	0.352	0.022	0.023	0.0224
Number of adult schistosomes	4.500 (±0.798)	4.000 (±1.118)	5.000 (±0.000)	4.375 (±0.924)	0.211	0.025	0.025	0.0246
Number of schistosome eggs excreted by humans	4.917 (±0.289)	4.222 (±0.667)	5.000 (±0.000)	4.667 (±0.565)	0.121	0.026	0.026	0.0259
Schistosome-contaminated water bodies	4.667 (±0.651)	4.556 (±0.527)	4.333 (±1.155)	4.583 (±0.654)	0.143	0.026	0.028	0.0266
Snail species (*Biomphalaria/Bulinus*)	4.833 (±0.389)	4.222 (±0.667)	4.333 (±1.155)	4.542 (±0.658)	0.145	0.026	0.025	0.0255
Snail density	4.917 (±0.289)	4.444 (±0.726)	4.333 (±0.577)	4.667 (±0.565)	0.121	0.026	0.025	0.0255
Miracidia infecting snails	4.583 (±0.515)	4.556 (±0.726)	4.000 (±1.732)	4.500 (±0.780)	0.173	0.025	0.025	0.0251
Snail lifespan	4.500 (±0.674)	4.000 (±1.000)	3.000 (±1.000)	4.125 (±0.947)	0.230	0.023	0.025	0.0239
Cercariae released by snails	4.667 (±0.651)	4.222 (±0.833)	4.333 (±1.155)	4.458 (±0.779)	0.175	0.025	0.024	0.0247
Cercariae infecting humans	4.833 (±0.389)	4.667 (±0.500)	4.000 (±1.732)	4.667 (±0.702)	0.150	0.026	0.025	0.0257
Human immunity	4.750 (±0.452)	4.111 (±0.928)	4.333 (±0.577)	4.458 (±0.721)	0.162	0.025	0.026	0.0254
Water exposure (infected)	5.000 (±0.000)	5.000 (±0.000)	5.000 (±0.000)	5.000 (±0.000)	0.000	0.028	0.027	0.0274
Outdoor defecation/urination	4.750 (±0.622)	4.667 (±0.500)	5.000 (±0.000)	4.750 (±0.532)	0.112	0.027	0.026	0.0262
Improved healthcare system	4.833 (±0.389)	4.667 (±0.500)	4.667 (±0.577)	4.750 (±0.442)	0.093	0.027	0.026	0.0265
National schistosomiasis control plans	4.917 (±0.289)	4.778 (±0.441)	5.000 (±0.000)	4.875 (±0.338)	0.069	0.027	0.027	0.0269
Dedicated funding	5.000 (±0.000)	4.667 (±0.500)	4.667 (±0.577)	4.833 (±0.381)	0.079	0.027	0.027	0.0270
Schistosomiasis surveillance system	4.917 (±0.289)	4.667 (±0.500)	5.000 (±0.000)	4.833 (±0.381)	0.079	0.027	0.026	0.0268
Diagnostic capacity	4.833 (±0.389)	4.778 (±0.441)	5.000 (±0.000)	4.833 (±0.381)	0.079	0.027	0.027	0.0270
Snail control	4.833 (±0.389)	4.444 (±0.527)	4.667 (±0.577)	4.667 (±0.482)	0.103	0.026	0.026	0.0260
Preventive chemotherapy (including MDA)	5.000 (±0.000)	4.556 (±0.726)	4.333 (±0.577)	4.750 (±0.532)	0.112	0.027	0.027	0.0266
Medication	5.000 (±0.000)	4.556 (±0.726)	4.000 (±1.000)	4.708 (±0.624)	0.133	0.026	0.027	0.0267
Health education	4.833 (±0.577)	4.667 (±0.500)	4.667 (±0.577)	4.750 (±0.532)	0.112	0.027	0.026	0.0264
WASH implementation	4.917 (±0.289)	4.667 (±0.500)	4.667 (±0.577)	4.792 (±0.415)	0.087	0.027	0.026	0.0264
Construction of water infrastructure	4.917 (±0.289)	4.667 (±0.500)	5.000 (±0.000)	4.833 (±0.381)	0.079	0.027	0.026	0.0267
Self-protective behavior	4.917 (±0.289)	4.556 (±0.527)	4.333 (±0.577)	4.708 (±0.464)	0.099	0.026	0.026	0.0264
Sanitary toilet usage	4.833 (±0.389)	4.667 (±0.500)	4.667 (±0.577)	4.750 (±0.442)	0.093	0.027	0.026	0.0265
Active healthcare seeking	4.833 (±0.389)	4.667 (±0.500)	3.333 (±1.155)	4.583 (±0.717)	0.156	0.026	0.026	0.0260
Medication adherence	4.917 (±0.289)	4.444 (±0.726)	5.000 (±0.000)	4.750 (±0.532)	0.112	0.027	0.026	0.0265

SD = standard deviation; WASH = water, sanitation and hygiene; MDA = mass drug administration.

**Table 8:** The feasibility weights for the influence and intervention indicators.

Indicator	Mean of Feasibility Scores (±SD)				Coefficient of Variation	Delphi Weight	Entropy Weight	Comprehensive Weight
	Zimbabwean Experts	Chinese Experts	Other International Experts	All Experts				
Primary indicators								
Influencing factors	4.167 (±0.718)	4.778 (±0.441)	3.667 (±1.155)	4.333 (±0.761)	0.176	0.511	0.510	0.5104
Intervention measures	4.167 (±0.937)	4.222 (±0.833)	3.667 (±0.577)	4.125 (±0.850)	0.206	0.489	0.490	0.4896
Secondary indicators								
Socioeconomic factors	3.750 (±0.754)	3.889 (±1.167)	2.333 (±0.577)	3.625 (±1.013)	0.280	0.157	0.166	0.1613
Natural environmental	4.167 (±0.835)	4.222 (±1.093)	3.000 (±1.000)	4.042 (±0.999)	0.247	0.175	0.165	0.1698
Biological factors	4.000 (±0.853)	4.333 (±0.866)	2.667 (±0.577)	3.958 (±0.955)	0.241	0.170	0.170	0.1703
Personal behavior	4.250 (±0.754)	4.333 (±0.707)	2.667 (±0.577)	4.083 (±0.881)	0.216	0.177	0.182	0.1793
Administrative-level interventions	3.833 (±1.193)	3.667 (±1.000)	3.333 (±0.577)	3.708 (±1.042)	0.281	0.160	0.162	0.1609
Individual-level interventions	3.750 (±1.422)	4.000 (±0.707)	3.000 (±1.000)	3.750 (±1.152)	0.307	0.162	0.155	0.1585
Tertiary indicators								
Economic resources	3.583 (±1.084)	4.222 (±1.093)	3.000 (±1.732)	3.750 (±1.189)	0.317	0.026	0.027	0.0265
Population density	4.167 (±0.718)	3.556 (±1.130)	2.667 (±1.155)	3.750 (±1.032)	0.275	0.026	0.027	0.0267
Remoteness of residence	3.917 (±1.379)	3.222 (±0.833)	3.333 (±1.528)	3.583 (±1.213)	0.338	0.025	0.025	0.0250
Accessibility of healthcare	4.417 (±0.900)	3.778 (±0.972)	3.667 (±1.155)	4.083 (±0.974)	0.239	0.029	0.026	0.0274
Accessibility of safe water	4.167 (±1.193)	3.667 (±1.000)	4.000 (±0.000)	3.958 (±1.042)	0.263	0.028	0.025	0.0263
Occupational factors	3.917 (±0.900)	3.556 (±0.726)	2.333 (±0.577)	3.583 (±0.929)	0.259	0.025	0.027	0.0261
Age	4.167 (±0.718)	3.444 (±1.333)	3.000 (±0.000)	3.750 (±1.032)	0.275	0.026	0.026	0.0259
Temperature	3.917 (±1.311)	3.222 (±1.641)	3.000 (±2.000)	3.542 (±1.503)	0.424	0.025	0.024	0.0241
Precipitation	3.083 (±1.505)	3.333 (±1.658)	2.667 (±1.528)	3.125 (±1.513)	0.484	0.022	0.022	0.0221
Distance to water bodies	3.833 (±0.937)	3.556 (±1.333)	3.333 (±2.082)	3.667 (±1.204)	0.328	0.026	0.026	0.0258
Schistosoma species (*S. mansoni*/*S. haematobium*)	4.083 (±1.084)	4.111 (±1.054)	3.667 (±0.577)	4.042 (±0.999)	0.247	0.028	0.027	0.0277
Lifespan of adult schistosomes	3.333 (±1.371)	3.111 (±1.616)	2.333 (±1.528)	3.125 (±1.454)	0.465	0.022	0.021	0.0213
Number of adult schistosomes	3.250 (±1.422)	4.000 (±1.118)	3.000 (±1.000)	3.500 (±1.285)	0.367	0.024	0.026	0.0250
Number of schistosome eggs excreted by humans	4.000 (±0.953)	4.111 (±0.782)	3.667 (±1.155)	4.000 (±0.885)	0.221	0.028	0.027	0.0277
Schistosome-contaminated water bodies	3.083 (±1.443)	3.444 (±1.333)	3.000 (±2.000)	3.208 (±1.414)	0.441	0.022	0.023	0.0225
Snail species (*Biomphalaria/Bulinus*)	4.083 (±0.996)	4.222 (±0.833)	3.000 (±1.000)	4.000 (±0.978)	0.245	0.028	0.027	0.0276
Snail density	3.583 (±1.240)	4.222 (±0.833)	2.667 (±0.577)	3.708 (±1.122)	0.303	0.026	0.026	0.0260
Miracidia infecting snails	3.333 (±1.155)	3.889 (±0.782)	2.000 (±1.000)	3.375 (±1.135)	0.336	0.023	0.025	0.0244
Snail lifespan	3.167 (±1.267)	3.889 (±0.928)	2.000 (±1.000)	3.292 (±1.233)	0.375	0.023	0.025	0.0241
Cercariae released by snails	3.583 (±1.505)	3.889 (±1.054)	2.000 (±1.000)	3.500 (±1.383)	0.395	0.024	0.023	0.0238
Cercariae infecting humans	3.167 (±1.267)	3.778 (±0.833)	3.000 (±1.000)	3.375 (±1.096)	0.325	0.023	0.026	0.0247
Human immunity	3.250 (±1.138)	3.556 (±0.726)	3.000 (±1.000)	3.333 (±0.963)	0.289	0.023	0.025	0.0240
Water exposure (infected)	4.250 (±0.965)	4.778 (±0.441)	3.667 (±0.577)	4.375 (±0.824)	0.188	0.030	0.028	0.0293
Outdoor defecation/urination	3.917 (±0.996)	4.111 (±0.782)	3.667 (±0.577)	3.958 (±0.859)	0.217	0.028	0.027	0.0273
Improved healthcare system	3.667 (±0.778)	3.556 (±0.882)	3.333 (±0.577)	3.583 (±0.776)	0.216	0.025	0.026	0.0256
National schistosomiasis control plans	4.083 (±0.793)	3.778 (±0.833)	4.000 (±0.000)	3.958 (±0.751)	0.190	0.028	0.027	0.0271
Dedicated funding	3.750 (±0.965)	3.667 (±1.225)	3.333 (±0.577)	3.667 (±1.007)	0.275	0.026	0.027	0.0262
Surveillance system	3.833 (±0.835)	3.889 (±1.167)	3.667 (±1.155)	3.833 (±0.963)	0.251	0.027	0.026	0.0263
Diagnostic capacity	4.167 (±1.030)	4.222 (±0.833)	4.333 (±0.577)	4.208 (±0.884)	0.210	0.029	0.028	0.0287
Snail density control	3.250 (±1.215)	3.444 (±1.130)	2.333 (±0.577)	3.208 (±1.141)	0.356	0.022	0.023	0.0228
Preventive chemotherapy (including MDA)	4.167 (±0.937)	4.333 (±1.000)	3.333 (±0.577)	4.125 (±0.947)	0.230	0.029	0.027	0.0279
Patient medication	4.083 (±0.669)	3.889 (±0.928)	4.000 (±1.000)	4.000 (±0.780)	0.195	0.028	0.027	0.0273
Health education	4.167 (±0.835)	4.222 (±0.833)	4.333 (±1.155)	4.208 (±0.833)	0.198	0.029	0.028	0.0285
WASH implementation	3.667 (±0.985)	4.222 (±1.202)	3.667 (±0.577)	3.875 (±1.035)	0.267	0.027	0.027	0.0267
Construction of water infrastructure	3.333 (±0.985)	3.333 (±1.000)	3.667 (±0.577)	3.375 (±0.924)	0.274	0.024	0.024	0.0240
Self-protective behavior	3.333 (±0.985)	3.889 (±0.782)	3.667 (±1.528)	3.583 (±0.974)	0.272	0.025	0.025	0.0251
Sanitary toilet usage	3.417 (±0.996)	3.667 (±1.000)	2.667 (±0.577)	3.417 (±0.974)	0.285	0.024	0.025	0.0244
Active healthcare-seeking behavior	3.417 (±0.996)	3.667 (±1.118)	2.333 (±0.577)	3.375 (±1.056)	0.313	0.023	0.024	0.0237
Medication adherence	3.417 (±1.311)	3.667 (±1.225)	3.000 (±1.000)	3.458 (±1.215)	0.351	0.024	0.024	0.0244

SD = standard deviation; WASH = water, sanitation and hygiene; MDA = mass drug administration.

## Data Availability

For access to the data obtained from public databases, please contact the corresponding author.
